# Text Typing Using Blink-to-Alphabet Tree for Patients with Neuro-Locomotor Disabilities

**DOI:** 10.3390/s25154555

**Published:** 2025-07-23

**Authors:** Seungho Lee, Sangkon Lee

**Affiliations:** 1 School of Future Technology, Korea University of Technology and Education, Choenan 31253, Republic of Korea; 2 School of Industrial Management, Korea University of Technology and Education, Choenan 31253, Republic of Korea; sklee@koreatech.ac.kr

**Keywords:** Lou Gehrig’s disease, amyotrophic lateral sclerosis, eye blinking, eye typing, neuro-locomotor disabilities

## Abstract

Lou Gehrig’s disease, also known as ALS, is a progressive neurodegenerative condition that weakens muscles and can lead to paralysis as it progresses. For patients with severe paralysis, eye-tracking devices such as eye mouse enable communication. However, the equipment is expensive, and the calibration process is very difficult and frustrating for patients to use. To alleviate this problem, we propose a simple and efficient method to type texts intuitively with graphical guidance on the screen. Specifically, the method detects patients’ eye blinks in video frames to navigate through three sequential steps, narrowing down the choices from 9 letters, to 3 letters, and finally to a single letter (from a 26-letter alphabet). In this way, a patient is able to rapidly type a letter of the alphabet by blinking a minimum of three times and a maximum of nine times. The proposed method integrates an API of large language model (LLM) to further accelerate text input and correct sentences in terms of typographical errors, spacing, and upper/lower case. Experiments on ten participants demonstrate that the proposed method significantly outperforms three state-of-the-art methods in both typing speed and typing accuracy, without requiring any calibration process.

## 1. Introduction

Amyotrophic lateral sclerosis (ALS), commonly known as Lou Gehrig’s disease, is an incurable disease that causes muscle weakness in the hands and feet, gradually progressing throughout the body [1,2]. It is caused by degenerative changes in the motor neuron system. The progress of degeneration and speed vary from patient to patient, but eventually, the entire body becomes paralyzed, and the patient usually lies in bed performing all activities there. Dysarthria makes communication difficult [3]. In the late stages of ALS, the patients can only move their eye muscles [1,4]. Accordingly, methods of communicating through eye movements and eye blinking have been utilized. The first type is that a letter board is directly shown to the patient, and the words are completed by inferencing the areas that the patient is looking at [5]. This method has the disadvantage that an assistant is required to hold the letter board next to the patient and interpret the patient’s gaze into letters. The second type is when the patient makes use of a digital tool called an eye tracker to analyze the position of the patient’s eyes and type the corresponding letters [6,7]. This type has the advantage that it can be used independently without an assistant. However, the equipment is relatively expensive, and there is a hassle in setting up the equipment because the calibration process must be performed to set the eye tracker according to the patient’s posture and eye movement. As mentioned in [8,9], the equipment that detects eye movements must be recalibrated if the body or chin moves because the eye movement and gaze point change.

In this paper, we propose a new method of eye gesture recognition-based typing to overcome the difficulties and inconveniences of patients with Lou Gehrig’s disease, as explained above. The main contribution is two-fold:

(1) We suggest a very intuitive typing method, the so-called Blink-to-Alphabet Tree (BAT), which gradually narrows down the range to the desired letter through eye blinks and grouped hierarchical letters. For example, if a patient wants to type the alphabet ‘B’, they first select the group ‘ABC/DEF/GHI’, then select the group ‘ABC’, and finally select ‘B’. Blinking of the right eye is used for moving the cursor between letters, and blinking of the left eye is responsible for selecting a group of letters. To customize this method for different patients, BAT can have modified hierarchical structures. For example, we could change the size of each group or expand the tree to allow for typing semantic group of letters (‘for’, ‘to’, ‘ing’, ‘tive’, and so on).
(2) To accelerate the eye gesture-based typing, LLM (large language model) [10,11] is also incorporated into the proposed method. Once the patient selects the ‘send’ button, the input words or sentence can be corrected in typo, spacing between words, and upper/lower cases, via OpenAI (Chat GPT) API [12]. As demonstrated in experiments, this could significantly reduce time for typing and improve accuracy.

The rest of the paper is organized as follows. In Section 2, we summarize the prior studies related to the proposed method. In Section 3, we describe the proposed method in detail. In Section 4, we present the experimental results, and Section 5 concludes the paper.

## 2. Related Works

Patients with Lou Gehrig’s disease eventually communicate mainly through eye gestures as muscle paralysis progresses. Communication methods based on eye gestures are generally divided into two categories, depending on the type of tool used to detect gestures like eye blinking as follows:

(1) EEG-based methods: A text typing method based on a brain–computer interface using Electro Encephalo Graphic (EEG) signals has been proposed, which is called virtual keyboard [13,14]. Eye blinks are recognized based on EEG signals to control the virtual keyboard. In [13,14], typing is performed by scanning one row of the keyboard at a time and selecting it with an eye blink, then scanning and selecting a group of three [13] or four [14] characters in the selected row and select with an eye blink, and finally choosing the desired alphabet character. This method has shown a high accuracy rate of about 95% [14], but it takes a long time to type because the patient has to wait for the desired group of characters to be displayed (e.g., it takes more than one minute to type five characters). In short, the EEG-based eye blinks method has limitations due to the difficulty of handling brain–computer interface (BCI) equipment, and it takes a long time to input the desired letters.
(2) Camera image-based methods: Some methods that rely only on camera images without any equipment (e.g., EEG) have also been proposed [7,15,16,17,18]. Eye tracker has been widely used to allow patients to use computers or input letters on their own [7]. When the patient is lying down and looking at a specific letter key on a separately mounted monitor, the eye position in the camera image is detected to type the letter. In this process, there is a hassle of calibration. In addition, if the position of the head is slightly off, typing errors could occur [7]. Similarly to the methods in [13,14], Attiah and Khairullah [15] have proposed a method in which letters are activated one by one on a keyboard, and the patient can input the desired letter by blinking when the desired letter is activated (note that the method in [15] uses only camera images (without EEG signals) for eye blink detection, different from the methods in [13,14]). This method has the advantage of being able to input words with simple blinking, but it is still inefficient in that the patient has to wait for the desired letter to be activated. Sushmitha et al. [17] have proposed a more systematic blink-based method, where Morse code was defined as a combination of short and long blinks. However, since all letters from A to Z must be represented by different blink patterns, the patient could feel a heavy burden in inputting letters. In [18], a simplified Morse code method has been proposed, which classifies the alphabet into four groups (‘A to G’, ‘H to N’, ‘O to U’, and ‘V to Z’) and significantly reduces the number of Morse codes. The patient first selects the group (e.g., ‘H to N’) by blinking twice in a row. Then, the Morse code for the desired letter within that group is input. At this time, the seven Morse codes defined can be shared and used in the same way in the four different groups, so the patient has fewer blink patterns to input, which reduces input errors. However, the Morse code-based typing method [16,17,18] requires a considerable amount of time to input letters. The proposed method can be more intuitive and less error-prone than existing blink-based methods because it groups the alphabet in a hierarchical structure and uses blinks for group navigation and selection in the visualized alphabet tree. The experimental results showed that the time required to input a sentence (i.e., ‘Change Body Position’) was about twice as fast as that of the comparison methods.

## 3. Text Typing with Blinks-to-Alphabet Tree

**Figure 1:**
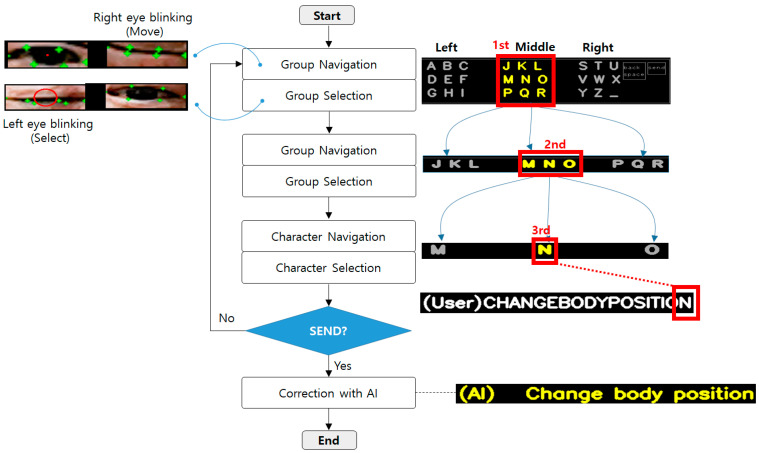
Flowchart of the text typing using the Blink-to-Alphabet Tree (BAT).

Figure 1 shows a flowchart of the text typing using the BAT. In this paper, the target characters are English alphabets letter which are commonly used worldwide. The proposed method aims to be usable even by patients in severe ALS conditions, such as those who cannot even move their heads and can only move their eye muscles. The existing text typing methods based on eye trackers require a very precise and repetitive calibration process to predict which character the patient is looking at. Because the patient’s posture or position could change slightly during the process, repetitive calibration can be burdensome. On the other hand, the proposed method simplifies the character selection process by limiting the text groups to three regions. For example, the number of candidate letters is narrowed down from 27 to 9 (1st to 2nd), 9 to 3 (2nd to 3rd), and 3 to 1 (1st to final decision). If the send button is selected, the set of words or the sentence entered by the patient is corrected by an LLM API. The corrections include fixing spaces, correcting upper/lowercase letters, and correcting typos. Table 1 shows the mapping table between the ternary value and the associated alphabet, which is used to select the alphabet to input through three sequential pairs of eye blinking (move and select).

### 3.1. Text Navigation and Selection

**Figure 2:**
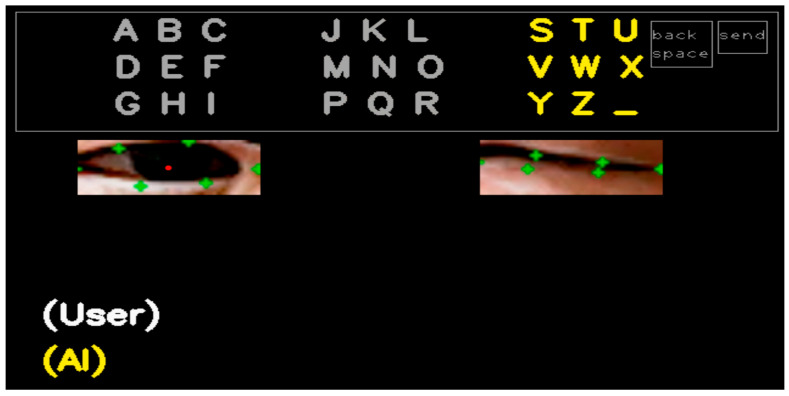
User interface for text typing with eye blinks.

**Figure 3:**
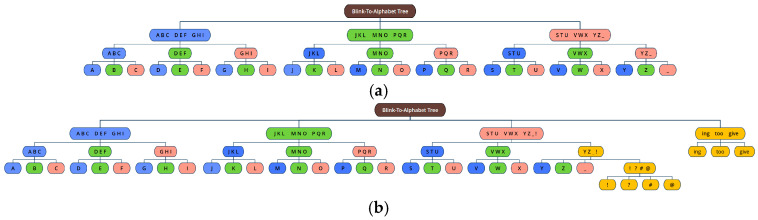
Blink-to-Alphabet Tree: (**a**) basic form; (**b**) a modified form.

In this section, we describe the details of typing letters using the BAT and eye blinks. Figure 2 shows user interface (UI) for text typing that is operated using blinks of the left eye and the right eye. There are three groups to navigate in the UI. In the first round, each group has nine letters, i.e., ‘A to I (left part)’, ‘J to R (middle part)’, and ‘S to Z and space bar (‘_’) (right part)’. Then, the selected group is split by rows and re-located such that S/T/U (left), V/W/X (middle), and Y/Z/_ (right). Finally, the selected group in the second round is again split by letters, for example, S (left), T (middle), and U (right), thus one out of the three alphabets is selected to type. In the lower part of the UI, the letters typed by the user are indicated sequentially (one by one) at the right part of ‘(User)’, as illustrated in Figure 1. In addition, the letters corrected by LLM are displayed at the right part of ‘(AI)’, which will be explained in Section 3.2. ‘Backspace’ is used to delete one letter, and ‘send’ is used for finishing the letter typing by the user and asking for correction by AI at the same time. Note that the ‘back space’ and ‘send’ buttons are only shown in the first round for efficiency during the typing process. Figure 3a shows the basic form of the BAT. The advantage of the proposed BAT is that it is very easy and simple to customize or expand according to the patients’ preference. For example, special characters or frequently used words (such as ‘ing’, ‘too’, ‘give’, etc.) can be placed at any position and depth, as shown in orange in Figure 3b.

**Figure 4:**
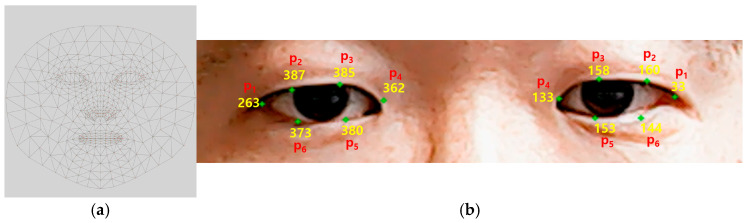
Illustrations of Mediapipe’s face landmark detection. (**a**) A total of 468 landmark points can be obtained through the face mesh solution. (**b**) A total of 12 landmark points can be selected for eye blink detections.

For the proposed method, the right eye’s blinking is used to navigate the text groups. More specifically, when the right eye is blinking repeatedly, it moves one step to the right, making the loop such that ‘A to I’, ‘J to R’, ‘S to _’, ‘back space’, ‘send’, and ‘A to I’ again. In addition, the left eye’s blinking is only used to select a text group (1st and 2nd rounds) or a letter (3rd round). Thus, three sequential selections can result in a single alphabet to type. For the purpose of eye blink detection, we use Mediapipe’s face mesh solution [19]. This solution first uses face detection, which is followed by a facial landmark detection in camera images. Out of the 468 points (see Figure 4a) the face mesh solution produces, we select 12 landmark points (6 for each eye) for measuring eye closeness, as illustrated in Figure 4b:

(1) Left eye landmark points: {263, 387, 385, 362, 380, 373}.
(2) Right eye landmark points: {33, 160, 158, 133, 153, 144}.

note that the left and right eyes can be easily distinguished by the indices of their landmark points. As the above landmark points are not {x, y} coordinates, we need to convert the relative positions in the output list of landmark points’ indices into the absolute coordinates.

**Figure 5:**
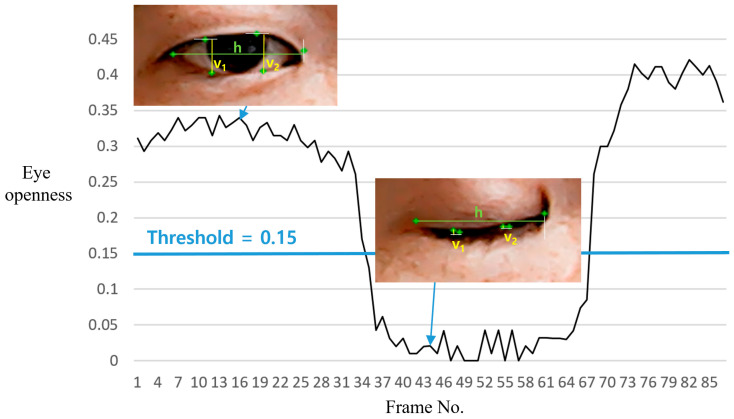
Illustration of changes in the eye openness (EO) measure over time.

**Figure 6:**
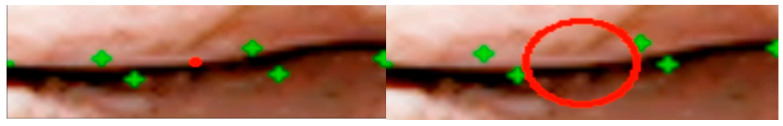
Visualization of cumulative ‘closed eye’ duration over time. The red dot (**left**) gradually grows into a circle (**right**), resulting in a letter selection to type.

Motivated by the Eye Aspect Ratio (EAR) [20,21], a simple but effective eye openness (EO) measure is used in this paper. As illustrated in Figure 5, EO is calculated as the ratio of the average vertical (y-axis) distance between the lower (p2, p3) and the upper points (p6, p5) to the horizontal (x-axis) distance between the inner (p4) and the outer (p1) points:

(1) $$EO=\frac{v_{1}+v_{2}}{2h},$$

where $h={abs(p}_{1}.x-p_{4}.x$), $v_{1}={abs(p}_{2}.y-p_{6}.y$), and $v_{2}={abs(p}_{3}.y-p_{5}.y$). Figure 5 clearly shows how the EO values vary according to whether an eye is closed or open. To detect eye blinks, EOs smaller than the threshold value are classified as ‘eye closed’. In this paper, the threshold is set to 0.15. As shown in the experiments, this setting works well for the ten volunteers. In the proposed method, a ‘closed eye’ state lasting for more than approximately 0.6 s is recognized as an intentional ‘eye blink’. The duration of a spontaneous eye blink is known to be between 0.1 and 0.4 s [22]. The mean duration of a blink is approximately 0.26 s, with a standard deviation of 11.3 ms [22]. Moreover, the proposed method utilizes single-eye blinks rather than bilateral blinks, enabling a clearer distinction between intentional and unintentional (spontaneous) blinking behavior. Therefore, a threshold of 0.6 could be sufficient for identifying intentional blinks, yielding consistent results across ten participants in the experiments. To enable the patient to intuitively perceive, when selecting a letter using the left eye blinking, a visualization is implemented on the UI, where the red dot gradually grows into a circle, as shown in Figure 6.

Note that our method uses a simplified version of EAR to define EO. For example, different from the method in [18], which uses Euclidean distance between landmark points, our proposed method uses the averaged distances along the vertical (y) and horizontal (x) axes, as illustrated in Figure 5. These measurements are more intuitive to interpret and require lower computational cost. Its validity has been demonstrated through experimentation. Furthermore, an intuitive visual cue, in which a red dot progressively enlarges into a circle, has been incorporated to assist in the detection of the patient’s blink input.

### 3.2. Words/Sentence Corrections Using LLM

**Figure 7:**
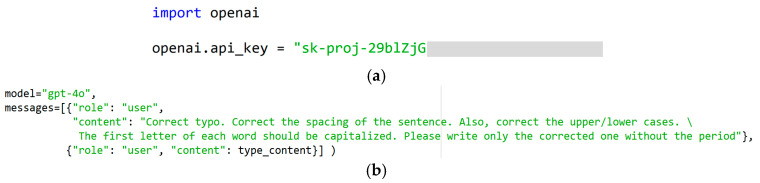
(**a**) Use of OpenAI API key in Python. Some characters of the API key have been masked for security reason. (**b**) The prompt used for the correction of words/sentence.

This section describes a method that can correct the text typed by the patient while also improving their typing speed by applying LLM to the proposed method. The BAT allows patients to correct their input words or sentences through an LLM when they select the ‘send’ button. For this purpose, we have obtained an OpenAI API key and utilized the API by importing OpenAI in Python (ver.3.9.13). An example of Python code for importing the OpenAI library and authenticating it with the API key is presented in Figure 7a. In this paper, GPT-4o is used as the chosen LLM. Three types of corrections are possible: typo correction, spacing correction, and uppercase/lowercase correction. For this purpose, the following was input into the prompt (refer to Figure 7b): “Correct typo. Correct the spacing of the sentence. Also, correct the upper/lower cases. The first letter of each word should be capitalized. Please write only the corrected one without the period.” In particular, even if the patient writes a sentence (e.g., CHANGEBODYPOSITION) without spaces and only in uppercase letters, it outputs a corrected sentence (e.g., Change Body Position), so it can significantly improve the typing speed.

It should be noted that, although the OpenAI API is employed for the LLM in this paper, offline LLMs can also be adopted in the proposed method without requiring an internet connection. This could offer advantages like enhanced privacy and the ability to avoid API usage limit and costs, which greatly improve usability.

## 4. Experiments

In this section, the experimental results of the proposed method are described in detail. Section 4.1 covers information on the participants and the experimental equipment setup. Section 4.2 describes the comparison results of text typing speed and accuracy with three recently proposed methods. Section 4.3 covers additional analysis results for the proposed method, including survey result analysis and effectiveness analysis of AI-based text correction.

### 4.1. Experimental Setup

**Figure 8:**
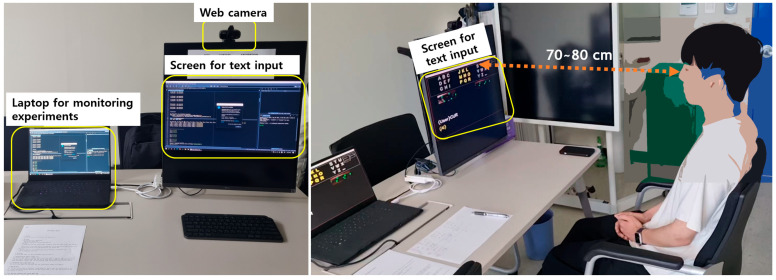
Experimental setup in the lab (**left**) and a participant during the experiment (**right**).

In this paper, the program of the proposed method was run on a laptop with Intel Core i7, NVIDIA GeForce RTX3080, and 32 GB RAM. A webcam model APC480 from ABKO was used, which has a specification of 640 × 480 pixels and 30 fps. A total of ten university students were selected as participants in the experiment. They were randomly selected to ensure fairness, resulting in seven male students, three female students, two eyeglass wearers, and eight non-wearers. Table 2 shows the information for the ten participants. Figure 8 shows the equipment setup in the laboratory and a participant in the experiment. The participants were asked not to move their upper body and head during character typing with eye gestures. To simulate conditions similar to neurodegenerative disorders, the participants were instructed to refrain from any intentional movement. However, because no facial fixation device such as a chin rest was used (as shown in Figure 8), some minor natural movement was allowed. Although it was difficult for non-patient participants to perfectly replicate the non-intentional periodic movements of a patient, we believe that the proposed method could work properly as long as the patients’ movements are not excessively large. The distance between the monitor and the participants was maintained at about 70~80cm.

### 4.2. Comparison Results with State of the Arts

In this section, we compare the character typing performance of the proposed method with three state-of-the-art methods. In [18], the authors made comparisons of their method with the remaining two methods [15,17] in typing speed and accuracy on a total of four English expressions (words or sentence). One of the limitations of implementing methods proposed by other researchers is that it is difficult to achieve optimal performance due to detailed parameter settings or environments. Hence, in this paper, we decided to directly cite the performance of the three methods reported in [18] by following the experimental conditions and procedures as closely as possible. First, we set the four patient intentions (PIs) frequently used by ALS patients as ‘Hot (PI-01)’, ‘Curtain (PI-02)’, ‘Absorption (PI-03)’, and ‘Change Body Position (PI-04)’. For each PI, the typing results (time required and accuracy) of the proposed method were averaged over the ten participants. Table 3 shows comparison results of the time required. In the method of [15], the users needed to wait for the desired letter to be activated before selecting it through blinking, thus it was inevitable that text typing took a long time. In the case of [17,18], it is understood that the methods took a relatively long time because they used a method of inputting Morse code-encoded alphabets through blinking patterns, which requires prior knowledge with Morse code or indexing the Morse code table while inputting. On the other hand, our proposed method could be more intuitive and faster than the methods above because the text navigations and selections relied on the visualized hierarchical tree structure (i.e., BAT) and the patient could simultaneously look at the character or character group to be selected. In addition, as explained in Section 4.3, for sentences (i.e., PI-04) with spaces, the improvement of typing speed via the AI correction is much more significant. The gaps of the typing speed became larger as the number of letters increased. Overall, the proposed method showed about twice the speed improvement compared to the three methods for the four PIs. Table 4 shows the details of the results for the proposed method in Table 3.

Table 5 shows compares three recent methods in typing accuracy. The advantages of our proposed method are also significant in terms of accuracy. Here, accuracy was calculated as {(total number of characters − number of typos)/total number of characters} × 100. Compared to methods that patternized the alphabets into Morse codes, in our proposed method, the patient directly sees and selects the characters with their eyes, which results in a lower probability of typos. Furthermore, AI correction could be successfully applied to minor typographical errors (for more details, refer to Section 4.3). As a result, except for PI-01, all of the participants were able to achieve 100% accuracy.

### 4.3. Further Analysis of Proposed Method

**Figure 9:**
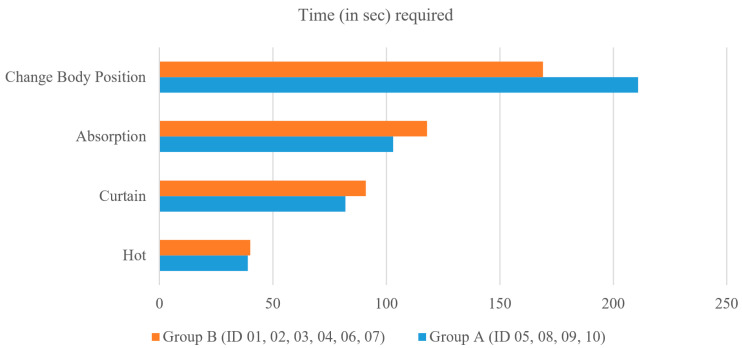
Comparisons of the text typing speed between Group A and Group B.

We further analyzed the effect of the LLM (AI correction) used in the proposed method. When the patient inputs their intended words or sentences and enters the ‘send’ button, those can be automatically corrected through OpenAI’s API, and the writing result can be saved; here, the time required for correction is only about 1 s. In the experiment, four participants (Group A: ID 05, 08, 09, 10) directly entered the space (‘_’) twice in ‘CHANGE BODY POSITION’. On the other hand, the remaining six participants (Group B: ID 01, 02, 03, 04, 06, 07) wrote ‘CHAGEBODYPOSION’ without any spacing. As shown in Figure 9, Group A took on average of 211 s, whereas Group B, using AI correction, took 169 s, resulting in a very large time difference of 40 s. An interesting result is that for the remaining PIs (i.e., Hot, Curtain, Absorption) without spacing, Group A took less time for typing. This result showed that LLM helped improve typing speed. During the experiment, there was a case where an AI correction was provided to fix a typo. ID 06 unintentionally misspelled ‘CURTAIN’ as ‘CURTAINJ’, but after pressing the ‘send’ button, it was corrected as ‘CURTAIN’. As a result, the accuracy of PI-02 was improved from 98.33% to 100.00% (see Table 5). Through the experiment, it was confirmed that corrections were possible for small fraction of misspelled letters among multiple letters.

**Figure 10:**
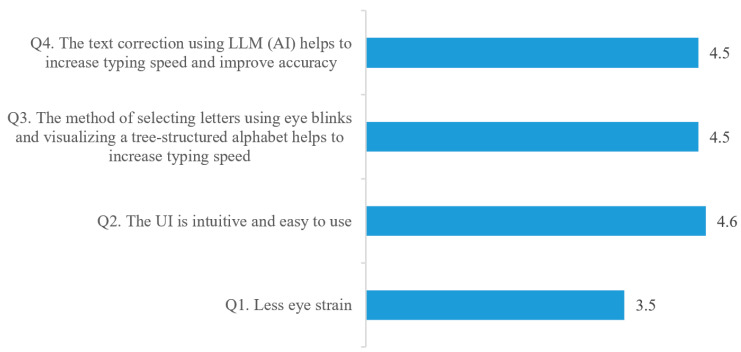
Results of the questionnaire for the proposed method, where ‘5’ is ‘most favorable’, and ‘1’ is ‘least favorable’.

For a qualitative evaluation of the proposed method, a survey was conducted after the experiment involving the ten participants. The survey used a 5-point Likert scale, where 1 point represented ‘least favorable’ and 5 points represented ‘most favorable’. The questions consisted of a total of four items, including eye strain (Q1), UI intuitiveness (Q2, Q3), and AI correction (Q4). As shown in Figure 10, for Q2, Q3, and Q4, we received positive evaluations with scores of 4.5 to 4.6 out of 5. This, together with the results from the comparison of typing time and accuracy in Section 4.2, demonstrates its effectiveness as a typing method. However, we received a relatively low score of 3.5 in terms of eye fatigue. This could be anticipated to some degree when considering the condition of a patient with ALS who was paralyzed and could only rely on eye gestures. To reduce discomfort and fatigue, it would be necessary to reduce the number of blinks further and to replace some gestures with appropriate movements of the eyeball. The proposed method is based on the capability to distinguish between blinks of the left and right eyes. However, it should be noted that some patients with progressive neuro-locomotor disorders, including ALS, may no longer retain the ability to control their eyes independently. To deal with this problem, an alternative way is to employ eye blinking for character selection and gaze detection for navigating the Blink-to-alphabet tree. For gaze detection, based on the patient’s gaze direction, eye positions are categorized into left, middle, and right, and this categorization can be readily used to facilitate text navigation, as illustrated in Table 1.

## 5. Conclusions

In this paper, we proposed a method for text typing based on eye gestures for rapid and accurate communication for patients with total paralysis, such as those with ALS. The proposed method utilized eye blinks to type texts intuitively with graphical guidance in the screen. Specifically, using a hierarchical tree-based UI called Blink-to-Alphabet Tree (BAT), the patient could input a desired alphabet via eye blinks, narrowing down the range through a number of sequential selections of text groups. The proposed method integrated an API of a large language model (LLM) to further accelerate text input and correct sentences in terms of typographical errors, spacing, and letter casing. To demonstrate the effectiveness of the proposed method, comparative experiments were conducted with ten participants, involving both quantitative and qualitative assessments. In the comparisons with three state-of-the-art methods, it was demonstrated that the proposed method achieved an improvement of 1.4 to 2.3 times in typing speed while maintaining a higher accuracy rate for the four patient intentions (i.e., Hot, Curtain, Absorption, and Change Body Position). At this time, we confirmed that text correction through LLM could potentially impact both accuracy improvement and typing speed enhancement. In the survey, the questions about the UI’s intuitiveness, the improvement in typing speed with the BAT and eye blinking, and the text correction through LLM received positive scores of 4.5 to 4.6 out of 5. However, in the question related to eye strain, the method scored relatively lower, with a score of 3.5 out of 5. For future work, we will conduct research to minimize the number of eye blinks or combine it with other eye gestures to reduce eye fatigue for the patients. Further directions for future research also include the following topics:

- Extend the study to real ALS patients: future work should validate the robustness and usability of the method with real ALS patients under real-world clinical conditions.
- Analyze robustness of EO under variable conditions by investigating how the eye openness (EO) metric behaves under varying lighting, camera angles, involuntary tremors, and user fatigue, as these can significantly affect blink detection reliability.
- Add multilingual support by evaluating how the method can be adapted for different languages (e.g., accented Latin letters, Cyrillic, Korean, etc.), which may require restructuring the tree or modifying character groupings in the BAT.
- Quantify eye blink detection accuracy by including performance metrics such as precision, recall, and false positive/negative rates for blink detection.

This study focused on conducting a comparative evaluation using eye blinking-based typing methods in camera images as a benchmark, with the aim of minimizing both cost and user inconvenience for patients even with severe paralysis. The authors believe that the proposed method could be further consolidated by incorporating a more sophisticated tool for eye blink detection. For example, a communication method based on eye-blinking detection using radar glasses [23] could be considered, given its robustness to the effect caused by unintended body motion and its privacy-preserving advantages.

## Figures and Tables

**Table 1:** Mapping of alphabets to three-digit ternary value.

3 Digit Ternary Value			Alphabet to Type
1st	2nd	3rd	
Left	Left	Left	A
Left	Left	Middle	B
Left	Left	Right	C
Left	Middle	Left	D
Left	Middle	Middle	E
Left	Middle	Right	F
Left	Right	Left	G
Left	Right	Middle	H
Left	Right	Right	I
Middle	Left	Left	J
Middle	Left	Middle	K
Middle	Left	Right	L
Middle	Middle	Left	M
Middle	Middle	Middle	N
Middle	Middle	Right	O
Middle	Right	Left	P
Middle	Right	Middle	Q
Middle	Right	Right	R
Right	Left	Left	S
Right	Left	Middle	T
Right	Left	Right	U
Right	Middle	Left	V
Right	Middle	Middle	W
Right	Middle	Right	X
Right	Right	Left	Y
Right	Right	Middle	Z
Right	Right	Right	spacebar (marked with ‘-’)

**Table 2:** Information for the ten participants.

ID No./Gender/Glasses	Left Eye Image	Right Eye Image
ID 01/Female/No	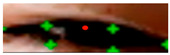	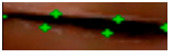
ID 02/Male/No	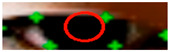	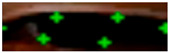
ID 03/Male/No	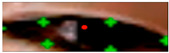	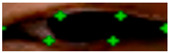
ID 04/Female/Yes	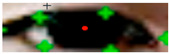	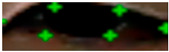
ID 05/Male/Yes	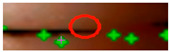	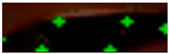
ID 06/Male/No	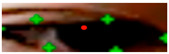	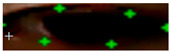
ID 07/Male/No	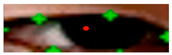	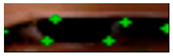
ID 08/Female/No	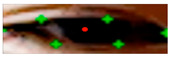	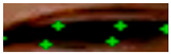
ID 09/Male/No	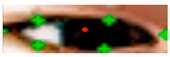	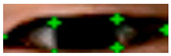
ID 10/Male/No	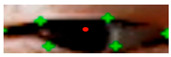	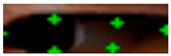

**Table 3:** Comparisons with three recent methods in terms of time required for typing (unit: s).

Patient Intention		[15]	[17]	[18]	Proposed
PI-01	Hot	82	62	57	40
PI-02	Curtain	196	176	132	87
PI-03	Absorption	256	220	173	112
PI-04	Change Body Position	418	425	342	186

**Table 4:** Results of ten participants for the proposed method in terms of time required for typing (unit: s).

ID No.	PI-01	PI-02	PI-03	PI-04
ID 01	36	112	112	202
ID 02	43	70	132	136
ID 03	39	133	126	220
ID 04	42	82	71	130
ID 05	39	77	100	171
ID 06	49	78	161	163
ID 07	30	69	108	162
ID 08	34	80	88	195
ID 09	37	96	95	237
ID 10	47	74	130	239
mean	**39.6**	**87.1**	**112.3**	**185.5**
Std. deviation	**5.5**	**19.6**	**24.5**	**37.2**

**Table 5:** Comparisons with three recent methods in typing accuracy. In order to verify the effectiveness of the BAT in the proposed method, the accuracies obtained without AI-based correction were also indicated.

Patient Intention (PI)		[15]	[17]	[18]	Proposed	
					Without AI Correction	With AI Correction
PI-01	Hot	80.00%	64.67%	93.00%	90.00%	90.00%
PI-02	Curtain	72.00%	61.14%	91.86%	98.33%	100.00%
PI-03	Absorption	63.70%	60.20%	93.50%	100.00%	100.00%
PI-04	Change Body Position	53.21%	44.05%	92.10%	100.00%	100.00%

## Data Availability

The data presented in this study are available on request from the corresponding author.
